# A Descriptive Review of Medication-Overuse Headache: From Pathophysiology to the Comorbidities

**DOI:** 10.3390/brainsci13101408

**Published:** 2023-10-01

**Authors:** Srdjan Ljubisavljevic, Marina Ljubisavljevic, Radomir Damjanovic, Sreten Kalinic

**Affiliations:** Department for Neurology, University Clinical Centre of Nis, 18000 Nis, Serbia; maraljub@gmail.com (M.L.); rdamjanovic@gmail.com (R.D.); kalinics@gmail.com (S.K.)

**Keywords:** medication-overuse headache (MOH), headache

## Abstract

Purpose of review: Medication-overuse headache (MOH) is an important problem worldwide, with different areas of controversy regarding its entity. This article reviews the risk factors, comorbidities, pathophysiology, clinical presentation, effective management, and prognosis of MOH by summarizing and integrating the results and findings from previously performed more than 15,000 studies (from 2010 to 2023) available from the scientific database of the University Medical Library in the University Clinical Center of Niš, which aimed to investigate and define the complexity of this type of headache. Recent finding: It has been proposed that all acute migraine medications can lead to MOH, with differences in the propensity of different agents to cause the problem. Early data suggests that triptans and other painkillers used for the acute treatment of migraine may be an exception. Recent studies show that practitioners and the general public are still largely unaware of the problem of medication overuse and its damaging effects. Summary: Although it is likely that MOH does occur, restricting the number of acute medications is necessary to prevent it. It is also possible that increasing amounts of acute medications are simply a reflection of poorly controlled headaches rather than a cause. Further research needs to be developed to identify more precise mechanisms for effective MOH management and its evolution.

## 1. Introduction 

Medication-overuse headache (MOH) is a secondary headache that is classified by the third edition of the International Classification of Headache Disorders [1] as a group of headaches attributable to the administration or discontinuation of various substances. MOH occurs more than 15 days per month in patients with preexisting headaches. It occurs as a result of regular (at least 3 consecutive months) overuse (10 or 15 days, depending on the type of medication) of drugs used as acute or symptomatic headache therapy [1].

### 1.1. Epidemiology

The estimated global prevalence of medication-overuse headaches (MOHs) is about 3%. MOH is one of the most common secondary headaches, affecting approximately 80 million people worldwide [2,3]. In tertiary care centers, 50% of headache patients suffer from MOH. This type of headache more commonly occurs in women (2:1 to 5:1) and typically occurs between ages 30 and 50 of life [4,5,6,7,8]. It can also occur in the pediatric population, but there are not many epidemiological studies that have approved that. MOH is more prevalent in urban areas (14.5% versus 2.1%) [9]. Some studies have shown that the prevalence of MOH is higher in people with lower socioeconomic status [10]. The prevalence of MOH is higher in patients receiving social assistance (11%), in people who have recently retired (7.5%), and in those on frequent absenteeism and sick leave (6%) [11]. A higher prevalence of MOH was also found in migrants [12,13,14]. Despite studies, there is no clear evidence of an association between lifestyle factors such as smoking, obesity, or physical inactivity and the development of MOH [15,16,17,18].

In terms of negative impact on daily, individual, and social activity, as well as the life quality of patients and their living and work environments, MOH is highly ranked. Measuring through years of life with disability (YLDs), MOH ranks 18th among all investigated causers [19,20]. The negative effects of MOH on the patient are far greater than the negative influence of migraine or tension-type headaches [21,22,23]. MOH has significant negative effects on education, career, monthly income, and social activities [24]. MOH is one of the most expensive headaches and the most expensive disease in general [25]. This burden of MOH could be prevented by early identification of patients with primary or secondary headaches who are at risk for developing MOH, which could provide more efficient treatment and a multidisciplinary approach to prevention.

### 1.2. Pathophysiology

**Figure 1:**
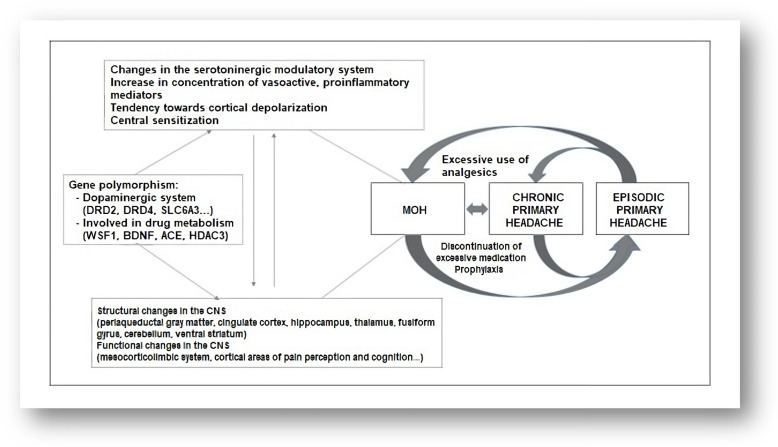
Pathophysiology of MOH.

**Figure 2:**
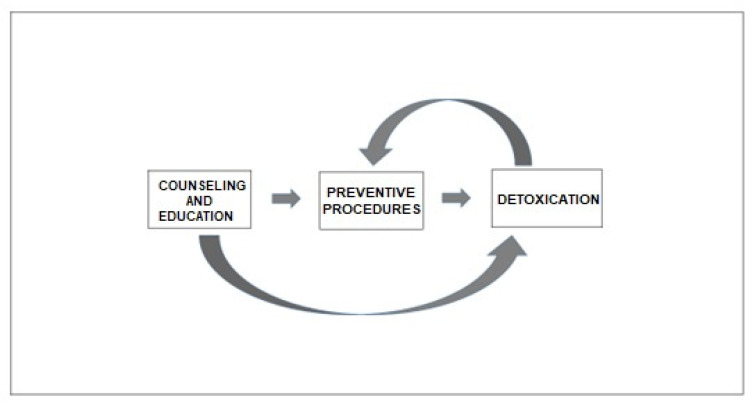
Algorithm for treating MOH.

The pathophysiology of medication-overuse headaches (MOHs) is not fully understood. In addition to factors related to previous headaches, psychological factors, personality traits, and a genetic predisposition play important roles [26,27] (Figure 1). Genes involved in complex processes of endogenous pain modulation, drug metabolism, and the serotonin and dopamine systems have been linked to the development of MOH [28]. Excessive use of analgesics leads to facilitation of supraspinal pain transmission [29] (Figure 2). Functional neuroimaging studies have revealed structural and functional abnormalities in various structures responsible for processing painful stimuli and in structures that provide the neurobiological basis of affective and addictive behaviors, such as the mesolimbic system [30] (Figure 1). While cessation of excessive medication use leads to a loss of these changes in some patients, they persist long-term in others. This is understood as a kind of predisposition to the development of medication overuse [31]. Experimental studies have shown that excessive use of medications to treat migraine leads to changes in descending pain modulation pathways and that these changes are associated with a decrease in serotonin levels and a decrease in the density of certain opioid receptors [32]. There are numerous experimental, genetic, structural, and functional neurovisualization and electrophysiological studies addressing the pathophysiology of medication-overuse headache (MOH). Experimental studies have shown that chronic exposure to sumatriptan leads to a reduction in the stimulus threshold and a sustained increase in the likelihood of cortical depolarization [33,34,35]. Increased production of CGRP, substance P, and NO in the trigeminal ganglion has been demonstrated in MOH [36,37], characterized by a reduction in the nociceptive threshold and inhibitory mechanisms [38]. Chronic use of analgesics leads to increased neuronal excitability in the amygdala complex, which may explain the occurrence of anxiety and depression in MOH [39]. In MOH, the activity of the serotonergic pain modulation system is impaired, with reduced production of serotonin in the CNS and increased expression of the pronociceptive serotonin receptor (5HT-2A) [40,41,42]. In animal models, increased expression of 5HT-2A receptors on platelet membranes and decreased serotonin concentration in these cells have been demonstrated with excessive use of analgesics [43] (Figure 2).

Genetic studies suggest the possible importance of polymorphisms in genes related to the dopaminergic system (DRD4, DRD2, and SLC6A3) and genes involved in the neurobiology of the addiction syndrome (WSF1, BDNF, ACE, and HDAC3) [11] (Figure 1). Central sensitization plays a key role in the pathophysiology of chronic migraine [44]. Analysis of somatosensory evoked potentials has shown that patients with chronic migraine have cortical hyperexcitability compared to healthy controls and patients with episodic migraine [45,46]. A follow-up study has observed that central sensitization is reduced with the cessation of overuse of medication [47]. Neuroimaging studies in patients with chronic migraine demonstrate increased gray matter volume in the periaqueductal gray region, posterior cingulate cortex, hippocampus, thalamus, fusiform gyrus, cerebellum, and ventral striatum [48]. Decreased gray matter volume has been observed in the orbitofrontal cortex, anterior cingulate cortex, occipital gyri, insula, and precuneus [49]. These structures are involved in endogenous pain modulation, cognition, and affective and addictive behavior [50]. Changes in white matter volume have been observed in the insular cortex and parietal operculum, but these findings have been inconsistent across different studies [51,52].

Functional neuroimaging studies have shown functional changes in regions involved in nociceptive processes, such as the mesolimbic reward system, the fronto-parietal system, and other regions [53,54]. The mesolimbic system consists of the ventromedial prefrontal cortex, the accumbens region, the substantia nigra, and the ventral tegmental area, which show altered functions in MOH. These findings have been associated with the occurrence of psychiatric comorbidities in MOH [55,56]. The described changes may be reversible after discontinuation of excessive medication [57]. Positron emission tomography (18F-fluorodeoxyglucose) has been used to demonstrate hypometabolism (during excessive medication use) in regions involved in pain processing, which returned to normal after successful discontinuation of excessive medication use with analgesic therapy. There was also a decrease in metabolic processes in the orbitofrontal region after the discontinuation of excessive drug use [58]. A decrease in gray matter volume in the orbitofrontal region was associated with a higher number of headache days per month after discontinuation of excessive medication use. Unsuccessful discontinuation of excessive medication use was associated with a reduction in the volume of the orbitofrontal region before discontinuation [59]. Successful discontinuation of excessive drug use resulted in a reduction in previously increased gray matter volume in the brainstem, whereas no such changes were observed with unsuccessful discontinuation [60]. Dysfunction of the ventromedial prefrontal cortex is potentially reversible and related to headache, whereas dysfunction of midbrain dopaminergic structures is long-term and likely related to excessive drug use [55,57]. The numerous structural and functional changes in the nociceptive system observed in the studies conducted suggest that there is a neurobiological basis for MOH (Figure 1).

### 1.3. Risk Factors

The risk of developing medication-overuse headache (MOH) is lower with excessive use of triptans and ergotamine than with combined analgesic therapy and opioids [58]. Another study showed that excessive use of barbiturates and opioids carries a two-fold higher risk of developing MOH compared with triptans and nonopioid analgesics. This study showed that nonsteroidal anti-inflammatory drugs (NSAIDs) may play a protective role in headache chronification in patients with a lower number of headache days per month. However, it has been shown that they may also accelerate chronification in patients with more headache days (>10 days) per month [59]. Although MOH can occur from any type of headache, it most commonly arises from the chronicity and transformation of migraine [60]. Patients with other painful conditions who are overmedicated for pain may not develop MOH unless they have had prior headaches [61,62]. In a prospective study involving 25.596 patients without chronic headaches, only 201 (0.8%) developed MOH 11 years later [63]. This study showed that risk factors for the development of MOH included the use of tranquilizers, the presence of other pain syndromes, mostly skeletal-muscular, the presence of gastrointestinal symptoms, the presence of depression and anxiety, physical inactivity, and smoking. Migraine and a higher number of headache days per month were found to be risk factors. Nonmodifiable risk factors include age (<50 years), female sex, and a lower education level. Some risk factors for chronic headache have also been identified (smoking and physical inactivity), but they lead to MOH only in association with overuse of analgesics [64]. A family history of MOH or substance abuse (alcohol, etc.) increases the risk of developing MOH threefold [65].

### 1.4. Type of Excessively Used Therapy

Excessive use of any type of analgesic therapy can lead to the development of medication-overuse headaches (MOHs) (Figure 2). Excessive use of triptans leads to a more rapid development of MOH compared to other analgesic therapies [66]. There is research evidence that suggests both triptans and nonsteroidal anti-inflammatory drugs (NSAIDs) can lead to MOH only when the frequency of previous headaches is increased. In fact, NSAIDs may have a preventive effect on patients who have fewer than 10 headache days per month and may prevent the chronicity of headaches [66,67]. Opioids and barbiturates increase headache chronification and the development of refractory headaches compared with triptans and NSAIDs [68]. Excessive medication use can lead not only to the worsening of headaches but also to other health problems. Chronic use of NSAIDs can lead to cardiovascular, renal, and gastrointestinal problems. Excessive use of acetaminophen can lead to liver damage. Opioids have sedative properties and may lead to the development of a withdrawal syndrome with the occurrence of autonomic phenomena upon sudden discontinuation of excessive medication. Barbiturates can cause epileptic seizures when excessive medication is suddenly discontinued. Excessive medication with benzodiazepines can lead to sedation and cognitive problems. For these reasons, gradual discontinuation of these agents is recommended. Signs of ergotism and peripheral arterial insufficiency [69] may occur in patients taking excessive ergotamine. MOH has several subtypes, depending on the type of medication taken excessively. All these data are summarized in Figure 1.

### 1.5. Psychosocial Factors

The presence of psychiatric comorbidities, certain personality traits, and socioeconomic status are considered important factors in the development of medication-overuse headaches (MOHs). Anxiety and depression are common in MOH patients compared to patients with episodic headaches and represent a risk factor for the development of MOH [70,71,72]. Anxiety, depression, stress, and a tendency toward a ruminative thinking style are present in MOH patients [73,74,75]. Psychological studies of MOH patients have shown that they often have difficulty controlling their emotions and behavior [70]. Obsessive-compulsive personality traits are common in MOH patients, while some MOH patients also exhibit addictive behavior [76,77]. MOH patients have been shown to be highly dependent on excessive medication use [78]. The presence of addictive behavior is a predictor of poor prognosis and poor treatment outcomes in MOH [79,80]. Introversion, decreased social activity, perfectionism, and dysphoric features are common findings in MOH patients [78,81]. High levels of psychological distress associated with poor lifestyle habits (smoking, physical inactivity, obesity) may be associated with the development of MOH [82]. Depression, anxiety, paranoia, and personality disorders may negatively impact the process of medication overuse cessation and treatment outcomes in MOH [83,84]. Socioeconomic factors are important for treatment outcome and the frequency of relapse in MOH. Unemployment, smoking, regular alcohol use, low socioeconomic status, immigration, and loneliness have a negative impact on MOH treatment outcomes [85,86].

### 1.6. Impact of Medication-Overuse Headache (MOH)

As mentioned previously, medication-overuse headache (MOH) has a significant negative impact on the daily and social activities and quality of life of affected individuals [87,88]. Studies analyzing differences between MOH patients have shown that these negative effects vary. In the COMOESTAS study, MOH patients who overused triptans and ergotamines had a lower score on the MIDAS questionnaire (fewer negative effects of headache) compared to MOH patients who overused opioids or multiple types of analgesics. In addition, this study showed that patients who overused triptans had fewer psychiatric comorbidities (depression, anxiety) [89]. It was observed that migraine patients with other pain syndromes, significant dysfunction, and activity limitations were more likely to overuse analgesics [90]. These findings improved the value of a multidisciplinary approach to MOH patients. Some patients overuse analgesic therapy out of fear of the negative consequences of the headache they have been suffering from for some time [91,92]. It has been shown that patients who continuously use excessive analgesic medication have a slightly better quality of life compared to those whose excessive medication is abruptly discontinued. This is interpreted as a direct effect of the excessive medication itself and suggests that the process of discontinuing the excessive medication may also have a negative effect on the patient [93].

**Figure 3:**
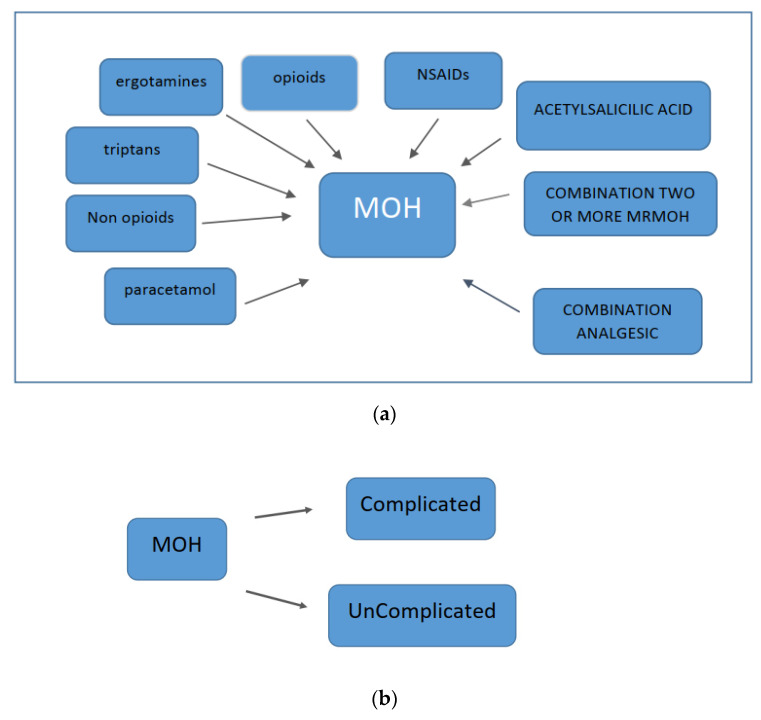
Types of MOH: (**a**) type 1 and (**b**) type 2.

MOH can be uncomplicated (type 1) or complicated (type 2) [94] (Figure 3).

An important factor that influences the complexity of CGRP-related disorders (CRDs) is the duration of the disease (>1 year), the presence of relapses, and previous unsuccessful attempts at therapeutic management [95]. Interpersonal differences between patients with CRDs in terms of clinical complexity and patient impact suggest possible different pathophysiological processes in different patients.

### 1.7. Comorbidities

Recognizing comorbidities in migraine patients is important to study their relationship, causality, shared etiology, pathogenesis, and other aspects. Psychiatric comorbidities are common, especially anxiety and depression [89,95]. In the COMOESTAS study, 40% of migraine patients met criteria for depression and 27.7% met criteria for anxiety [96]. In the EUROLIGHT cross-sectional study conducted in 10 countries of the European Union, similar results were obtained, with a greater difference in the frequency of these comorbidities compared to the group with migraine without excessive use of analgesics [97]. The SAMOHA study examined the frequency of psychopathologic comorbidities in migraine patients compared to patients with episodic migraine and healthy controls [98]. The frequency of moderate to severe anxiety was higher in both groups with headaches, whereas the frequency of addictive disorders was significantly higher in patients with migraine. Patients with migraines often had multiple psychiatric comorbidities. Obsessive-compulsive syndrome has been shown to be associated with migraine [99]. One-third of migraine patients may have subclinical forms of obsessive-compulsive syndrome, which is a recognized risk factor for the chronicity of migraine [100,101]. Migraine may be associated with behavioral disorders related to substance use [72,102,103,104,105,106,107,108,109,110,111,112]. Although similar neurobiological mechanisms are hypothesized for these two entities, it has been shown that there are distinct personality traits in patients with migraine and those with addiction syndromes [113,114]. Obesity is a risk factor for the chronicity of migraine, although there are studies that have questioned this association [114]. An association has been demonstrated between obesity, physical inactivity, smoking, and the occurrence of migraine [97]. The association between obesity and migraine has also been observed in children [115]. Patients with migraines are more likely to have sleep disturbances [116].

## 2. Diagnosis

Medication-overuse headache (MOH) occurs for 15 or more days per month and develops as a result of regular excessive use of medication for acute symptomatic headache (10 or more or 15 or more days per month, depending on the type of medication) for at least 3 months (Table 1). The exact number of days after which use of the corresponding medication is considered excessive is based on expert recommendations [117].

A patient with preexisting primary headache who develops a new type of headache or a marked worsening of a preexisting headache in temporal relation to excessive medication use should have both criteria for MOH (one or more subtypes, see Figure 1) and a diagnosis of preexisting headache [117].

One patient may have multiple subtypes of MOH. For example, a patient who meets the criteria for triptan overuse headache and one of the subtypes of simple analgesic overuse headache should have both diagnoses. In the case of overuse of combination analgesics, the diagnosis of combination analgesic overuse headache is made rather than the diagnosis of overuse of each component of the combination analgesic. There is a possibility of frequent use of different types of medications for acute/symptomatic headaches that, based on the total number of days taken, meet the criteria for excessive medication use, although no single medication or group of medications is used excessively. These patients are diagnosed with MOH attributable to multiple classes of medications. Patients who overuse multiple medications for the treatment of acute/symptomatic headaches but do not provide specific details about these medications in detail are diagnosed with MOH, which is attributed to the unverified overuse of multiple classes of medications. On average, half of patients who have headaches for15 or more days per month for a period of more than 3 months also have MOH [118].

### 2.1. Headache fromExcessive Use of Ergotamine

Due to the variable bioavailability of ergot preparations, it is difficult to define a minimum dose or number of days of this therapy that must be met to diagnose this headache [1].

### 2.2. Headache fromExcessive Use of Triptans

Excessive use of triptans can increase the frequency of migraines and lead to their chronification [118].

### 2.3. Headache Due to Overuse of Common Analgesics

This headache most commonly arises due to overuse of paracetamol, acetylsalicylic acid, and nonsteroidal anti-inflammatory drugs [118].

### 2.4. Headache Due to Overuse of Opioids

It has been demonstrated that patients who excessively used opioids had the highest degree of headache relapse following detoxification therapy [118].

### 2.5. Headache Due to Overuse of Combined Analgesics

The term “combined analgesic” is used for a specific formulation that combines two or more classes of drugs, each with analgesic properties or acting as an adjuvant. There are many combined analgesics that are widely used among patients, and they are often overused. The most commonly overused combined analgesics combine common analgesics with opioids, butalbital, and/or caffeine [118].

### 2.6. Medication-Overuse Headache Attributed to Multiple Classes of Drugs, Rather Than Individual Overuse

Fulfillment of criteria without overuse of any individual drug or drug class indicates that criterion B is not fulfilled for any of the previously mentioned types of medication-overuse headaches (ergotamines, triptans, common analgesics, opioids, and combined analgesics) [118].

### 2.7. Medication-Overuse Headache Attributed to Unverified Overuse of Various Classes of Drugs

Patients who overuse multiple drugs for the treatment of acute or symptomatic headache but cannot provide accurate information about what, when, or how much they take are common in clinical practice. Daily monitoring/recording for several weeks is of particular importance, but it should not delay treatment of MOH if diagnostic criteria are met [118].

## 3. Therapy

The ideal treatment for medication-overuse headaches (MOHs) is the subject of numerous debates. Since there is a diverse spectrum of clinical manifestations and interindividual differences among patients, the ideal treatment for MOH should be individually tailored. Treatment for MOH should begin in the outpatient setting, with patient education, discontinuation of medications for excessive use, and initiation of transient or long-term preventive therapy. This approach may be effective in two-thirds of patients, whereas in the remaining patients, the effect is unsuccessful [119].

Some studies have compared the effectiveness of outpatient discontinuation of excessive medication (without additional therapy) with hospital-assisted discontinuation using detoxification strategies. The group consisted of patients with MOH without prior detoxification attempts and without significant psychiatric comorbidities. No differences in treatment success were observed between these groups (75% success rate in both groups) [85]. A favorable treatment outcome for MOH was recorded in patients who received brief education about MOH and its treatment in primary healthcare. These patients did not receive additional preventive therapy. After 6 months, satisfactory results were achieved in two-thirds of the patients [120]. So-called brief interventions (educations) have also shown a favorable long-term effect, with a relapse rate below 10% after 16 months [120,121]. When comparing the discontinuation of excessive medication in outpatient and hospital settings with the introduction of preventive therapy in both groups, no significant difference was observed in initial success after 2 months (58% outpatient vs. 37% hospital) or in long-term success after 2 years (44% outpatient vs. 40% hospital) [122]. Better treatment outcomes and a lower relapse rate in discontinuation of excessive medication in outpatient settings compared to hospital settings have been observed in some prospective studies [123]. This approach is rational for uncomplicated MOH, as it is cost-effective and may be more comfortable for the patient and their environment. The decision to adopt this approach is based on an assessment of the patient’s compliance. In the process of discontinuing excessive medication, particularly during the follow-up period, the primary healthcare physician may be involved. Access to healthcare and counseling services is significant in preventing the relapse of MOH after achieving immediate satisfactory effects of medication discontinuation [124]. Support in the form of work absence can be beneficial in the process of discontinuing excessive medication [125]. In the case of excessive medication with barbiturates, benzodiazepines, and opioids, the presence of psychiatric comorbidities, and previous attempts at discontinuation, it is advisable to conduct MOH treatment in hospital settings. Conducting discontinuation in hospital settings allows for the application of parenteral therapy and control of analgesic intake. Day hospitals can be a useful alternative to the hospital model for discontinuation of excessive medication [126]. Abrupt discontinuation of excessive medication is a better strategy for excessive use of non-opioid analgesics such as triptans and ergotamine. Gradual discontinuation is recommended for excessive use of barbiturates, benzodiazepines, and opioids due to the possibility of withdrawal syndrome associated with these substances [127,128]. Abrupt discontinuation is better tolerated by conducting so-called bridging therapy. Abrupt discontinuation of analgesics and ergotamine in hospital settings with acute analgesic therapy can have long-term beneficial effects in a significant number of patients, even up to 5 years after discontinuation [129]. Although both abrupt and gradual discontinuation of excessive medication led to a reduction in disability and an improvement in quality of life, abrupt discontinuation has proven to be more effective [130,131]. With abrupt discontinuation, a worsening of headaches can be expected. During withdrawal in MOH, prednisone reduces rescue medication without decreasing the severity and duration of withdrawal headaches. Discontinuation headaches typically last for 4 days after triptan discontinuation, 7 days after ergotamine discontinuation, and up to 10 days after discontinuation of other analgesics [130]. Withdrawal syndrome from barbiturates and opioids can last up to 10 days (with an average duration of 3.5 days) and typically involves symptoms such as nausea, vomiting, headache, anxiety, restlessness, sleep disturbances, and an increased heart rate [132]. The use of clonidine (0.1–0.2 mg/day) is beneficial for suppressing autonomic symptoms following opioid discontinuation [133]. Phenobarbital is useful in suppressing potential seizures after discontinuing barbiturates [130].

There is not enough research examining the preventive effects of pharmacotherapy on the occurrence of MOH in patients with episodic headaches who are at increased risk for developing MOH. Topiramate has shown a significant effect in preventing episodic migraine from progressing to chronic migraine, compared to placebo, in a follow-up period of 6 months with a significant number of patients (756 patients) [134]. Topiramate leads to a reduction in the number of headache days per month and a decrease in the need for symptomatic analgesic therapy. However, a number of patients, even with topiramate, still develop chronic migraine and MOH. There are not enough quality conclusions to support the use of preventive pharmacotherapy in preventing the transformation of episodic migraine into chronic migraine and the development of MOH [135].

### 3.1. Bridging Therapy

This therapy prevents the onset of withdrawal headaches. The use of naproxen, which has a relatively long duration of action compared to other NSAIDs, is beneficial for treating headaches during the process of medication overuse withdrawal [132]. Low doses of tizanidine (2–16mg), a central alpha-2 adrenergic receptor agonist, in combination with NSAIDs are effective in the medication overuse withdrawal process in two-thirds of patients [132]. The use of corticosteroid therapy has shown partial effectiveness. There was no demonstrated greater efficacy of oral prednisolone compared to placebo in the medication overuse withdrawal process [136].

There was no demonstrated superiority of intravenous methylprednisolone or paracetamol compared to placebo in controlling withdrawal headaches [132]. Meta-analysis did not show the effectiveness of prednisone in reducing the number of headache days during medication overuse withdrawal compared to placebo [132]. The use of neuroleptics such as chlorpromazine and prochlorperazine may be beneficial due to their significant antidopaminergic effect in pain control [133]. However, the use of these medications should be limited to hospital settings due to possible side effects such as sedation, extrapyramidal symptoms, and cardiovascular symptoms. Parenteral administration of dihydroergotamine is effective in controlling headaches due to medication overuse withdrawal [135]. The use of dihydroergotamine in the medication overuse withdrawal process is long-term effective in preventing the occurrence of refractory headaches [135]. The parenteral administration of valproic acid has limited effectiveness. The use of aspirin is beneficial in the process of medication overuse withdrawal. Intravenous administration of lidocaine significantly prevents the onset of headaches and has long-term beneficial effects [137]. This approach requires cardiovascular monitoring [126,137,138,139]. Infiltration of the greater occipital nerve origin with lidocaine and corticosteroids is useful and safe [140].

### 3.2. Discontinuation of Medications Overused Medications and Preventive Therapy 

Discontinuation of medication overuse is the cornerstone of the therapeutic approach for patients with MOH. A significant number of patients achieve satisfactory results and do not require additional preventive therapy after medication overuse withdrawal [141]. Abrupt withdrawal of medication overuse without additional preventive therapy leads to a significant reduction in the number of headache days in slightly less than half of the patients [142]. Significant improvement has been observed in patients with MOH who have been overusing triptans and ergotamines. There was no significant difference in the success rate of medication overuse withdrawal between patients who were not on preventive therapy and those who used preventive therapy during the withdrawal process [142]. In a follow-up study of patients with complicated and uncomplicated MOH who received counseling treatment without preventive therapy but with headache support treatment (paracetamol, antiemetics), after 14 weeks, 92% of patients with uncomplicated MOH and 65% of patients with complicated MOH were free of medication overuse. A significant percentage in both groups (80% and 57%) showed improved functionality [143]. After initial short-term education about MOH, after one and a half years, 42% of the surveyed patients with chronic headaches had a reversal to an episodic course of headaches, while 76% of the patients reduced their excessive use of analgesics [144].

Discontinuation of excessive medication use before initiating preventive therapy is advised in several previous guidelines for the treatment of MOH [145,146]. The use of analgesic therapy during the discontinuation period is recommended to prevent and alleviate withdrawal symptoms [147,148]. In the COMOESTAS study, 46% of participants achieved a reversal from chronic to episodic headaches and successfully discontinued medication overuse using a protocol that limited but did not prohibit the use of acute analgesic therapy [147]. The efficacy of a protocol involving abrupt discontinuation of excessive medication use without permission for acute therapy was compared with a protocol that allowed limited use of analgesic therapy for 2 days per week. It has been shown that abrupt discontinuation was more effective in reducing the monthly number of headache days (42% vs. 22%), but not significantly different in reducing the monthly number of days with acute analgesic use (reduction from 21.2 to 6.9 days per month with abrupt discontinuation and from 22.1 to 9.3 days per month with permitted limited use of analgesics) [149]. This effect was sustained after 12 months, although greater efficacy was observed in the group with abrupt discontinuation and prohibition of acute therapy. The percentage of long-term discontinuation efficacy ranges from 46% to 77% [150,151,152]. This percentage is between 40% and 50% in patients with refractory MOH [148]. High rates of successful discontinuation, 80–90%, have been reported in other studies [149,153]. Discontinuation is considered more effective than most other preventive therapies [150]. The use of preventive therapy in parallel with the discontinuation of excessive medication use leads to a 44% reduction in headache frequency during the first month, with an increase in the rate of frequency reduction to 59% after 6 months [151]. In 68% of these patients, a reversal to episodic headache type was observed, accompanied by significant reductions in depression and anxiety and improvements in quality of life.

Neuromodulation represents a new strategy for the treatment of MOH. High-frequency repetitive transcranial magnetic stimulation of the dorsolateral prefrontal cortex, compared to a placebo group, had a limited effect on the success of discontinuing excessive medication use [152]. Some studies find that the success of complete discontinuation of excessive medication use is sustainable even after several years but is emphasized by the use of additional preventive therapy [154]. It has been observed that successful discontinuation of excessive medication use also leads to a reduction in depression and anxiety [67]. There are studies indicating that a complete cessation of excessive medication is not necessary if preventive therapy is initiated. The use of topiramate in patients with chronic migraine transformed into MOH has shown effectiveness in reducing the monthly number of headache days [155]. The three-month effects of valproic acid compared to a placebo were evaluated in patients with MOH after short-term detoxification therapy. The success rate was significantly higher with valproic acid (45%) compared to the placebo group (23.8%) [155].

In studies of onabotulinum toxin A use in the prevention of chronic migraine (PREEMPT 1 and PREEMPT 2), two-thirds of the patients had MOH [156]. After two rounds of treatment, a significant reduction in the score on the Headache Impact Test (HIT) questionnaire was observed [119]. These patients did not undergo the so-called “traditional” withdrawal of excessive medication. Onabotulinum toxin A demonstrated good tolerability and effectiveness in a 12-month study of patients with chronic migraine transformed into MOH [115]. Treatment with onabotulinum toxin A leads to a reduction in the number of days with analgesic therapy after 3 months in patients with MOH [119]. 

The efficacy of monoclonal antibodies in the therapy of chronic migraine with and without MOH was examined [152]. The effectiveness of this therapy has been demonstrated in patients with chronic migraine and MOH without the need for traditional withdrawal of excessive medication. This effectiveness has been particularly shown for erenumab [152] and fremanezumab [154]. It has been observed that the use of fremanezumab leads to a significant reduction in the monthly number of days with analgesic therapy and a decrease in the frequency of moderate to severe headaches compared to the placebo group. These patients did not undergo a prior withdrawal of excessive medication [154]. The use of erenumab leads to a significant reduction in the monthly number of headache days by more than 50% and a decrease in the average monthly number of days with analgesic therapy, as well as a reduction in the HIT score, after 3 months of follow-up. In this study, 47% of the patients met the criteria for MOH [154]. There are studies assessing the effectiveness of cannabinoids, pregabalin, occipital nerve stimulation, and acupuncture in the treatment of MOH. However, the results of these findings do not have clinical significance. Although amitriptyline is used for the prevention of chronic migraine, there is no clear evidence of its superiority in the treatment of MOH. Considering the prevalence of depression in MOH, amitriptyline could be particularly useful as an antidepressant in selected patients with MOH. Additional non-pharmacological methods (such as relaxation techniques, etc.) can enhance the proven efficacy of topiramate, onabotulinum toxin A, and monoclonal antibodies targeting CGRP [126]. The use of beta-blockers and flunarizine can be considered in the treatment of MOH, but there is no clear evidence of their safe effectiveness [126]. The complexity of using preventive therapy is significant for the sustainability of the effect of medication withdrawal. Tolerability and a better profile of adverse effects of preventive therapy are considered crucial for the long-term use of this treatment [126].

### 3.3. The Treatment of Medication Overuse

The treatment of medication overuse syndrome can be pharmacological or non-pharmacological. The availability of healthcare professionals and psychological support are crucial for successfully overcoming withdrawal syndrome. Monitoring metabolic parameters, blood pressure, administration of parenteral therapy, sedation, and other symptomatic treatments are recommended. The use of dihydroergotamine, antidopaminergic therapy (chlorpromazine, prochlorperazine, metoclopramide, and droperidol), valproic acid, ketorolac, magnesium, or corticosteroids is advised [139]. There are suggestions for the use of tizanidine for this purpose [139]. During detoxification in MOH caused by barbiturates, benzodiazepines, or opioids, the use of long-acting opioids, phenobarbital, and clonidine is beneficial [8]. In cases of medication overuse not caused by opioids, symptomatic therapy is recommended for the first week of withdrawal. Triptans are advised for overuse of analgesics, and non-specific analgesics are recommended for overuse of triptans. Concurrently with this therapy, the use of metoclopramide (10 mg, i.m., p.o.), chlorpromazine (25–50 mg, i.m.), prochlorperazine (10 mg, p.o., i.m.), domperidone (30 mg rectal or 10 mg, p.o.), or levopromazine (6–25 mg, p.o., parenteral) is useful [8]. The use of paracetamol (1000 mg orally or intravenously) or naproxen (500 mg orally) is recommended as analgesic therapy for a maximum of 3 days during the first week of withdrawal. Parenteral administration is more effective. Some studies have compared the effectiveness of celecoxib (400 mg daily for the first 5 days with a reduction of 100 mg every fifth day until complete discontinuation) versus prednisone (75 mg daily for the first five days with a gradual dose reduction every fifth day until complete discontinuation) in the process of medication overuse withdrawal [8]. Corticosteroids (at least 60mg of prednisone or prednisolone) and amitriptyline (up to 50mg) are possibly effective in the treatment of withdrawal symptoms [157]. Celecoxib showed greater efficacy in controlling headache intensity; there was no difference in the frequency of headaches or the need for additional symptomatic therapy between these groups [8]. The use of prednisone (60 or 100 mg daily for five days) was not superior to placebo in controlling withdrawal headaches [8]. In these studies, corticosteroid use was associated with a lower need for additional symptomatic therapy compared to the placebo group. The use of methylprednisolone (500 mg, i.v.) and acetaminophen (4 g daily, i.v.) was not superior to placebo during the detoxification process [158].

For chronic migraine patients, the CGRP monoclonal antibody was slightly better than botulinum toxin in terms of efficacy and safety [126]. A double-blind experimental study was performed on adult patients with MOH who attended Sina Hospital in Tehran from June 2009 to June 2011. Greater occipital nerve (GON) block was performedin the two groups of patients by administering a combination of 3 mL of 0.5% bupivacaine and 2 mL of 5% saline or 3 mL of 0.5% bupivacaine and 80 mg of methyl prednisolone.

### 3.4. Patient Education and Multidisciplinary Care

MOH is considered a preventable headache disorder. Risk factors for the development of MOH include female gender, frequent prior headaches, frequent use of analgesic medications, inadequate control of previous headaches, use of benzodiazepines, smoking, physical inactivity, obesity, addictive behavior patterns, psychiatric comorbidities, other painful conditions, and lower socioeconomic status [15,16,17,18]. Healthcare professionals can identify patients with headaches who are at risk for developing MOH. However, the presence of these risk factors does not necessarily guarantee the occurrence of MOH, and modifying them can be challenging. The association between risk factors and MOH may not be direct but mediated by other factors, further complicating prevention efforts. Awareness of MOH among the general population, patients, medical students, pharmacists, and clinicians is inadequate [139]. Increasing awareness of MOH is considered a crucial step in preventing the chronicization of previous headaches and the development of MOH. A national awareness campaign conducted in Denmark increased awareness of MOH from 31% (pre-campaign) to only 38% [131]. The preventive effect of non-pharmacological strategies in preventing MOH has been evaluated. In patients with migraine, the preventive effect of education through brochures was compared to education through direct contact with healthcare professionals (training in coping strategies, etc.) regarding the development of MOH. None of the patients in either group developed MOH during the follow-up period, starting with an average of 22 headache days per month and an average of 7 days of analgesic use per month. A minimal transformation rate from episodic to chronic headache was observed [131]. Therefore, raising awareness of MOH among the general and professional populations is crucial for preventing medication overuse, headache chronicization, and the development of MOH. Education is an effective preventive strategy. Patients with episodic headaches at risk for developing MOH should be regularly monitored by primary care physicians and neurologists (every 3–6 months) [4]. Education about the necessity of discontinuing excessive medication use is effective in the treatment of MOH. In fact, this approach alone led to the transformation of MOH into an episodic headache form in up to 40% of patients [126]. The best strategy for prevention and treatment is education for every patient with headaches, as no completely certain factors for the transformation from episodic headache to MOH have been identified thus far. The availability of analgesic therapy is an additional risk factor for the development of MOH. The use of caffeinated beverages also contributes to headache chronicization and the development of MOH [159]. This education should be conducted at the primary healthcare level. Patients should be made aware of the recognized risk factors for the development of MOH, such as excessive use of analgesics (number of days per month), benzodiazepine use, the presence of anxiety/depression, gastrointestinal issues, physical inactivity, and smoking [64,82].

A study conducted by Norwegian authors demonstrated that brief counseling interventions have a significant effect on the prevention of MOH and reverting it to an episodic headache pattern in up to 50% of patients (in comparison to a group that did not receive counseling) [151]. In a study comparing the effect of the counseling approach to additional pharmacological interventions (such as steroid therapy, parenteral symptomatic and antiemetic medications, and other preventive therapies) in the treatment of MOH, it was observed that the counseling approach had a similar effect to preventive pharmacotherapy, with a reversal of episodic headaches and discontinuation of excessive medication use achieved in over 70% of patients. The study was conducted in a tertiary center and did not include patients with previous withdrawal attempts, psychiatric comorbidities, or excessive use of opioids and barbiturates [157].

Comparing the effectiveness of counseling interventions for discontinuation of excessive medication use in outpatient versus inpatient settings, no significant differences were observed [160]. In a long-term follow-up study assessing the effectiveness of counseling interventions in the treatment of chronic headaches and MOH, a decrease in the number of days with excessive medication use was observed, reducing from 22 days per month (at the beginning of the study) to 6 days per month, with 76% of patients no longer exceeding the recommended medication use. The study included patients with a mean duration of chronic tension-type headache (CTTH) ranging from 8 to 18 years and patients with a mean duration of MOH ranging from 5 to 10 years [161]. In another study, the effectiveness of MOH therapy in outpatient settings was investigated, which involved introducing preventive therapy, intravenous bridging therapy (using dihydroergotamine), and optimizing acute headache treatment. The impact of this approach on physical and psychological well-being was assessed over a longer period. The results showed a significant reduction in headache intensity and a decrease in the Headache Impact Test (HIT) score from 66 to 55 at the end of the follow-up period, along with a significant reduction in the levels of depression and anxiety during the follow-up [119]. Counseling interventions themselves are often adequate and effective in the treatment of MOH caused by triptans and non-opioid analgesics in the absence of significant psychiatric comorbidities. It should be implemented by healthcare professionals, including primary care physicians, neurologists, and specially trained healthcare providers. However, this approach is insufficient for patients with MOH due to excessive use of opioids, benzodiazepines, and barbiturates, as well as those with a history of unsuccessful attempts to discontinue excessive medication use. These patients require referral to specialized headache treatment centers [8]. Patients with medication-overuse headaches (MOHs) have a problem that extends beyond the headache itself. Psychological support during in-hospital medication overuse discontinuation has been associated with lower relapse rates and greater effectiveness in discontinuing excessive medication [97]. Occupational and psychotherapy, psychiatric and physical therapy have shown effectiveness [98]. Multidisciplinary management is effective even in patients with previous unsuccessful attempts at discontinuation. In a follow-up study, after 12 months of combined analgesic therapy following discontinuation of excessive medication, over 80% of patients no longer had MOH, with half of them experiencing a reduction in the number of headache days by more than 50%. Motivation from healthcare professionals improves the success rate of medication overuse discontinuation in adolescents with MOH [99]. Cognitive-behavioral therapy is beneficial for achieving greater efficacy in discontinuing excessive medication. Relaxation training, attention training, physical exercise, lifestyle and dietary changes, stress management, and biofeedback are useful methods in the treatment of MOH [100]. 

### 3.5. Clinical Monitoring and Prognosis

Clinical monitoring with access to a headache diary is necessary. Ideally, personal contact with the patient should be provided. Electronic diaries are considered significant in this process [162]. In cases of unsuccessful attempts at discontinuing excessive medication, additional therapeutic strategies should be considered. For the treatment of headache cessation, medications with a longer half-life and bioavailability should be chosen to reduce the need for re-administration of the therapy.

Therapeutic goals for MOH include reversing chronic headaches to episodic headaches, reducing the burden on daily functioning activities, and achieving headache control with the lowest possible amount of medication. Treatment of MOH has direct economic benefits for the healthcare system, leading to increased patient productivity and reduced indirect costs (reduced absenteeism) [137]. The success rate of discontinuing excessive medication varies [48]. In adolescents and children, multidisciplinary treatment of MOH has a higher success rate, including complete cessation, transitional therapy, and preventive therapy [75,139,156]. A meta-analysis of 17 studies involving 1101 patients with MOH found a 72% success rate of discontinuation after 6 months, along with improved quality of life and reduced disability [48]. The relapse rate of MOH ranges from 20% to 50% in most studies [151].

The follow-up period for assessing discontinuation success ranges from 6 months to 9 years. The relapse rate after 6 months ranges from 0% to 41% [66,87,95,143]. The relapse rate after 12 months ranges from 13% to 41% [141,163]. The use of onabotulinum toxin A, with or without other preventive therapies, has shown no relapses of MOH after 2 years [119,146]. During a 6-year follow-up period, the relapse frequency ranged from 21% to 45% [147,148]. After 9 years of follow-up, the relapse rate was 32% [145]. The relapse rate did not significantly differ across the follow-up periods [147]. Relapses most commonly occur within a year of discontinuation [148]. The following factors have been associated with increased relapse frequency: type of headache (more frequent relapses in cases of coexisting migraine and tension-type headache compared to migraine alone), type of excessively used therapy (rare relapses with excessive use of triptans) [11], frequency of migraine before and after discontinuation [11], use of multiple previous preventive therapies [120], unsuccessful discontinuation attempts in the previous three years, use of acute therapy in the emergency department after discontinuation, depression smoking, and alcohol use [131,132,133]. Predictors of successful and long-term discontinuation include successful discontinuation from the beginning and low headache frequency within a year of discontinuation [11]. Counseling with patients is significant. The relapse rate of MOH in patients who received counseling at the beginning of detoxification was 4% after 6 months and 8.3% after 16 months. Patients who received counseling 6 months after discontinuation experienced no relapse during the observed period [88]. The relapse rate in multidisciplinary treatment of MOH after previous unsuccessful attempts is 15% [163]. Combined pharmacotherapy and psychotherapy have shown superiority over pharmacotherapy alone in the process of discontinuation. This effect was sustained after Altieri [142]. Cognitive-behavioral therapy in combination with pharmacotherapy did not demonstrate superiority over pharmacotherapy alone in the discontinuation process, neither after 6 nor after 12 months of follow-up. Patients with migraine as the underlying cause of MOH have a higher relapse rate after one year compared to patients with tension-type headaches [86]. It has been shown that the relapse rate is lower with excessive use of triptans compared to combined analgesics, although there are studies that do not find such an association [121,142]. Identifying risk factors for MOH relapse after previous discontinuation is necessary and requires monitoring of all patients with MOH for an extended period. Monitoring the use of analgesic therapy after discontinuation is effective in evaluating the therapeutic success of detoxification and the occurrence of relapse. Psychotherapy is an effective additional method for the treatment of MOH and relapse prevention.

## 4. Personal Experience

In a study examining depression, anxiety, stress, tendency toward rumination, and quality of life in patients with newly diagnosed MOH compared to healthy controls, a total of 164 individuals were included, 33 males and 131 females, ranging in age from 18 to 65 years. The healthy control group consisted of 81 individuals, 22 males and 59 females, aged 20 to 65 years. The MOH group comprised 83 individuals, 11 males and 72 females, aged 18 to 65 years. The average duration of MOH prior to diagnosis was 5.5 years, with the majority of patients experiencing migraines (64%) with an average duration of less than 8 years before MOH development, mostly episodic in nature in over 90% of cases. Half of these patients used over-the-counter analgesics for acute migraine therapy, achieving partial therapeutic effectiveness. The average number of days with MOH per month was slightly over 19 days. Slightly more than half of the patients used combination analgesics for MOH therapy. Female gender, lower level of education, and marital status (married) were more common in MOH compared to healthy controls. The absence of alcohol consumption habits and lower levels of physical activity were more common in MOH [121,122,123].

The results of this study mostly confirmed the hypotheses on which the research was based. Moderate levels of depression, anxiety, and stress were present in patients with MOH. A higher degree of rumination was observed in MOH. Impairment of physical, mental, and overall health was present in MOH. Selective dependence of these parameters on the examined sociodemographic, clinical, and laboratory findings was observed. The degree of anxiety and rumination style of thinking were predictors for the occurrence of MOH, while the degree of depression and stress were not predictors for the occurrence of MOH. The degree of depression (along with the female gender) was a risk factor for the physical aspect of health in MOH. The degree of depression was a risk factor for mental and overall health in patients with MOH. The degree of anxiety, stress, and rumination style of thinking were not predictors of quality of life in MOH [121,122,123].

**Figure 4:**
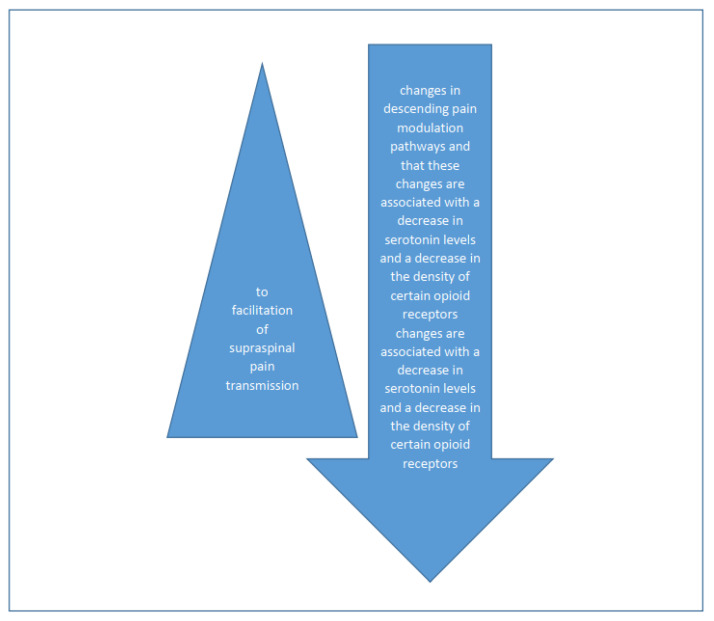
Functional neuroimaging studies have revealed structural and functional abnormalities in various structures responsible for processing painful stimuli and in structures that provide the neurobiological basis of affective and addictive behaviors, such as the mesolimbic system.

Based on the results of this study, it is concluded that assessing the degree of anxiety and rumination style of thinking in patients with chronic headaches could be useful for preventing the transformation of chronic headaches and the development of MOH. On the other hand, assessing the degree of depression in patients with MOH and its treatment would be beneficial for improving the quality of life of patients with MOH. The results indicate the necessity of a multidisciplinary, individualized approach to patients with chronic headaches and MOH. The goal of such an approach would be to prevent the occurrence of MOH and provide more effective treatment and a better quality of life for patients with MOH (Figure 4).

## 5. Conclusions

MOH is a secondary headache disorder, although pathophysiologically and clinically it represents a complication or transformation of the most commonly occurring primary headaches. It is a complex entity with biological, psychological, and social aspects. Assessing the risk factors for MOH occurrence and treatment success is crucial for the primary and secondary prevention of this type of headache. The diagnosis of MOH is clinical, and its treatment is individualized and highly dependent on a multidisciplinary team approach (neurologist, pain medicine specialist, behavioral psychologist, etc.) and should probably be attempted in primary care (leaving the more complicated cases to a neurologist specialized for this type of complexity). For those who suffer from MOH and have poor information about MOH management, a detoxication and prevention program should be chosen based on their clinical condition and evidence of intervention effectiveness. Discontinuation of excessive medication should be carried out abruptly for those who excessively use common analgesics, ergot preparations, and triptans. Gradual discontinuation is advised for excessive use of opioids, barbiturates, and sedatives. In addition, it can be carried out on an outpatient basis or in a hospital setting (the effectiveness is similar in all the conditions mentioned). In spite of this, the type of MOH is a crucial factor in selecting the location and method for discontinuing excessive medication. All crucial conclusions are presented in Table 2 and Table 3.

Either the discontinuation of medication overuse, transitional therapy, preventive therapy, preventive relapse monitoring, or education and the patient’s awareness are the foundations of the therapeutic approach. All crucial conclusions are presented in Table 2 and Table 3.

In spite of all that was mentioned above, MOH is a treatable and preventable public problem worldwide. The gain from treating patients with MOH is potentially high and may lead to substantial economic savings for society as well as for individual patients.

General conclusions and recommendations on the management of MOH are presented in Table 2 and Table 3. 

## Figures and Tables

**Table 1:** Subtypes of MOH.

Headache due to excessive use of ergotamines. Headache due to excessive use of triptans. Headache due to excessive use of non-opioid analgesics. Headache due to excessive use of paracetamol (acetaminophen). Headache due to excessive use of acetylsalicylic acid. Headache due to excessive use of nonsteroidal anti-inflammatory drugs (NSAIDs). Headache due to excessive use of opioids. Headache due to excessive use of combination analgesics. Headache due to excessive use of medication is attributed to multiple drug classes rather than individual excessive use. Headache due to excessive use of medication attributed to unverified excessive use of different drug classes. Headache due to excessive use attributed to other medications.

**Table 2:** General conclusions on the management of MOH.

Patient education is crucial for the management of MOH. Patients with uncomplicated MOH can be effectively managed in primary care. Patients with complicated MOH should be managed by a multidisciplinary team (neurologist or pain medicine specialist, behavioral psychologist, etc.). For patients with poor education on MOH management, a detoxification and prevention program should be chosen based on their clinical condition and evidence of intervention effectiveness. Discontinuation of excessive medication should be carried out abruptly for patients who excessively use common analgesics, ergot preparations, and triptans. Gradual discontinuation is advised for excessive use of opioids, barbiturates, and sedatives. Discontinuation can be carried out on an outpatient basis, through a day hospital, or in hospital settings. The effectiveness is similar in all the conditions mentioned. The type of MOH is crucial in selecting the location/method for discontinuing excessive medication.

**Table 3:** General conclusions about MOH.

MOH is a secondary headache disorder, although pathophysiologically and clinically, it represents a complication of the most commonly occurring primary headaches. The diagnosis of MOH is clinical. MOH is a complex entity with biological, psychological, and social aspects. Assessing the risk of MOH occurrence and treatment success is crucial for the primary and secondary prevention of MOH. Treatment for MOH is individualized. Discontinuation of medication overuse, transitional therapy, preventive therapy, monitoring to prevent relapse, prior education, and patient awareness are the foundations of the therapeutic approach. A multidisciplinary approach is necessary.

## Abbreviations

cAMP	Cyclic adenosine monophosphate
CDS	Color Doppler sonography
cGMP	Cyclic guanosine monophosphate
CGRP	Calcitonin gene-related peptide
CO	Carbon monoxide
COX-2	Cyclooxygenase-2
CRP	C-reactive protein
MOH	Medication-overuse headache
GTN	Glyceryl trinitrate
GTT	Tension-type headache
HIT	Headache impact test
MIDAS	Migraine disability assessment score
ICHD	International classification of headache disorders
MR	Magnetic resonance
MRA	Magnetic resonance angiography
MRV	Magnetic resonance venography
MSG	Monosodium glutamate
NO	Nitric oxide
NSAIDs	Nonsteroidal anti-inflammatory drugs
PACAP	Pituitary Adenylate Cyclase-Activating Polypeptide
PDE	Phosphodiesterase
ESR	Erythrocyte sedimentation rate SUNA, the short-lasting unilateral neuralgiform headache attacks with cranial autonomic symptoms
SUNCT	Eng. Short-lasting unilateral neuralgiform headache with conjunctival injection and tearing
TAG	Trigeminal Autonomic Headache and Trigeminal Neuralgia
